# Injection drug use in an affluent beachside community in Sydney: An exploratory qualitative study

**DOI:** 10.1111/dar.13592

**Published:** 2022-12-20

**Authors:** George Christopher Dertadian, Theresa Caruana, Lisa Maher

**Affiliations:** ^1^ Centre for Crime, Law and Justice UNSW Sydney Sydney Australia; ^2^ Centre for Social Research in Health, UNSW Sydney Sydney Australia; ^3^ Kirby Institute, Faculty of Medicine UNSW Sydney Sydney Australia

**Keywords:** affluent, injection drug use, middle‐class, qualitative research, stigma

## Abstract

**Introduction:**

Social research on injection drug use has focussed on marginalised groups and communities, leaving a large gap in the field's understanding of how it is experienced in other settings, including in relatively affluent communities.

**Methods:**

This research is based on fieldwork and 18 in‐depth qualitative interviews conducted in suburban beach‐side communities in Sydney collectively known as the Northern Beaches.

**Results:**

Participants did not experience stigmatisation by local health services as the norm or as a deterrent to access. Drug acquisition on the Northern Beaches occurred among closed networks of friends and acquaintances, and injecting use rarely occurred in public settings. Police contact was minimal, resulting in lower levels of criminalisation.

**Discussion and Conclusions:**

Unlike many of the participants featured in the literature, our study participants grew up in middle and upper middle‐class households, typically experiencing comfortable childhoods with little to no exposure to injection drug use. In this setting injection drug use operates covertly within the normal rhythms of middle‐class life, hidden in amongst the bustle of cafés and shopping centres, and through the friendliness of neighbourhood driveway and doorstep interactions. Drug use is described as common in the area, with injecting behaviours stigmatised in ways that set it against the ‘good’ families and neighbourhoods of this beach‐side enclave. In contrast to much of the Australian qualitative literature which frames injection drug use as a means of psychological relief or a subcultural norm, our participants described injecting as motivated by the desire to enhance pleasure and social connection.

**Key point**

- Study participants grew up in middle and upper middle‐class households with little to no exposure to injection drug use.
- On the Northern Beaches of Sydney injection drug use operates covertly within the normal rhythms of middle‐class life.
- In contrast to much of the Australian qualitative literature, our participant described injecting as motivated by the desire to enhance pleasure and social connection.

## 1 INTRODUCTION

The public image of injection drug use is one of destructive and dangerous urban pleasure‐seeking reified by images of discarded needles in the gutters and alleyways of the outskirts and underbellies of busy cityscapes. Layered into this image is the social disadvantage of those who are seen to lose themselves to the chemical seduction believed to characterise drug dependence and injection drug use [1, 2]. A propensity towards criminality, particularly property crime, is thought to be the inevitable result of a collision between disadvantage and drug use. This popular construction influences social and qualitative research: the task of the researcher becomes entangled with conventional discourse about injection drug use as a practice of the dangerous urban underclass [3]. This often results in research that focusses on the inner‐city where drug treatment and other social services are concentrated, as well as in outer suburban areas with high levels of social disadvantage. In these accounts injection drug use becomes framed as a form of psychological relief from trauma and marginalisation, or as a community norm.

This literature has produced valuable contributions to the field, with research firmly demonstrating that marginalisation from drug‐related stigma makes people more vulnerable to health and social harms. For example, in environments where visible drug use, poverty and social disorder are common, the prevalence and impacts of illicit and injecting drug use have been found to be high [4, 5, 6, 7]. These environments are also more likely to have a concentration of homelessness, unstable housing and public injecting, which are associated with increased risk of HIV and hepatitis C transmission [8, 9, 10, 11]. Furthermore, social research has illustrated how aggressive policing of street drug markets can increase the harms associated with injection drug use [12] and that this is exacerbated in ethnic minority communities [13]. More broadly, evidence also indicates that marginalised groups are criminalised by contact with police, including Indigenous people [14, 15], ethnic and cultural minority groups, migrant communities [16], people with disabilities [17] and young people [18, 19]. In its focus on the way drug stigma causes harm and marginalises people who use drugs, pleasure is conspicuously absent from the conversation [20, 21].

While these are valid areas of research, this means that the bulk of literature is about marginalised groups and communities, leaving a large gap in the field's understanding of how drug use and, for our purposes, injection drug use, occurs in other settings, including in relatively affluent communities. Little is known about drug use, injecting behaviours and related harms among people who are not socially and structurally disadvantaged and who have low levels of police contact and surveillance. This study aimed to address this gap by conducting in‐depth interviews with people who inject drugs in the affluent suburbs of Sydney collectively known as the Northern Beaches, and colloquially referred to as ‘The Beaches’.

## 2 METHODS

Between May and December 2020, the first author conducted interviews (*n* = 18) with people who inject drugs in the Northern Beaches of Sydney. The Northern Beaches is a region that covers approximately 30 km of the Eastern coastline of New South Wales, and extends inland to include the large 14,000 ha Ku‐ring‐gai Chase National Park. The beach‐side suburbs that make up the area were recently (2016) amalgamated from the Warringah, Manly and Pittwater Councils. During the study the first author was based in the field 2 days a week for 6 months, including 1 day based at the local needle and syringe program (NSP). Fieldwork also included spending time at local shops and shopping centres, parking lots and transport hubs. Observations in the field were not recorded as data, nor formally subject to data analysis. However, informal notes were taken about observations and conversations that occurred during fieldwork, which informed critical reflection on the field‐context, approaches to recruitment and engagement with participants [22, 23].

Participants were recruited to the study through a range of methods, including through flyers posted on community message boards and at local bus stops (*n* = 8), referrals from previous participants (*n* = 6), introductions by local NSP staff (*n* = 3) and a single referral from a local psychologist. The research took an inductive approach, consistent with established traditions in qualitative drug research that conduct ‘in‐depth analysis of relatively small samples of people who use drugs’ in order to provide ‘in‐depth understanding of the practices of particular groups of people who use drugs’ [24, 25]. Our project was concerned with providing a deep understanding [26, 27] of the experience of injection drug use among people who live in an affluent area, a group and setting we know little about.

Potential participants were screened by the first author using the following eligibility criteria: aged 18 years or older; self‐identifying as growing up in and currently living in the Northern Beaches; and self‐identifying as a person who injects drugs (defined as injection drug use in the past 10 years). The study's inclusion criteria were purposefully open in order to facilitate exploratory research in an under‐researched sub‐population. Interviews lasted between 30 and 90 min and were audio‐recorded and transcribed verbatim in tandem with data collection. Interview transcripts were analysed by thematic content through a combination of preliminary and close readings.

Preliminary analysis was conducted at the paragraph level and was guided by the questions asked in interviews. Close reading and thematic analysis [28] was then conducted by the first and second author to generate themes consistent across the questions asked. Following Braun and Clarke, our analysis is the ‘product of deep and prolonged data immersion, thoughtfulness and reflection’ [29, p. 591], an iterative process of continually reflecting on what is known in the field about injecting drug use, service access and criminal justice contact, and how this compared to the experiences of participants in our study. Critical reflection on the content of the interviews during fieldwork [30] by the first author also helped to hone and refine the interview probes and reflection during data analysis helped to identify and consolidate themes across the transcripts. In this way, the development of themes was purposeful, identifying significant elements of the lived experience and meanings of injection drug use among participants.

Ethical approval for the study was received from the UNSW Sydney Human Research Ethics Committee and participants were remunerated $AUD50. All interview participants nominated or were allocated a pseudonym to be used in any dissemination of study results.

## 3 RESULTS

The social position of the sample, made up of 11 men and 7 women, were distinct from the characteristics of samples presented in most qualitative social science research. The median age of participants was 48.5 years (range 24–67 years). The majority of the sample were Anglo‐Australian (*n* = 11), with the remainder identifying as first and second‐generation European migrants (*n* = 6), and one participant identifying as Aboriginal.

Heroin or pharmaceutical opioids were the main drugs injected by participants, although some participants also used other substances, such as methamphetamines and benzodiazepines. Most participants noted that when they first started injecting they quickly fell into a pattern of frequent (usually daily) injecting. The vast majority also indicated that their initiation to injecting did not take place on The Beaches, and most often occurred when they lived in inner‐city Sydney.

Most participants noted that they had gone through periods of fluctuating frequency of drug use, as well as periods of not using at all. Having children was the most common reason for longer periods of abstinence, especially among the women in the sample. For many participants (*n* = 8) it was normal to go weeks without injecting—indeed, several participants were not injecting at the time of interview or had gone weeks without doing so. Other participants currently injected a few times a week or less (*n* = 7), and a smaller group reported injecting daily (*n* = 3) at the time of interview. Many spoke about their drug use as motivated by the desire to enhance pleasure and social connection, and in ways that emphasised responsible consumption.

*“I've been pretty safe. Luckily I'm not silly, I'm not greedy with the heroin…* [I will inject] *every fortnight, you know what I mean, I'll ‘treat myself’ as you say”* (Pam 46‐year‐old female).

*“I prefer to have a little bit, put it down, go and do a few things. I'm not a pig”* (Jessie, 41 year‐old female).

*“*[I inject] *at home if my son's at work, and he's going to be at work for a long time. Because I don't do it in his face”* (Sandra, 50‐year‐old female).

Drug use was described as common and condoned within social circles, with higher incomes perhaps enabling more ‘functional’ use.

*“It's quite easy to find people on The Beaches to use* [with]*, especially when you're young. If you grow up here, especially if you come to places like* [this]*. If you go into places like* [the] *mall or any local shopping centre, you'd meet a group of people in a subculture that use drugs”* (Seb, 30‐year‐old male).

*“Rich people, you know, beach people… they're more laid back and surfies I suppose. And they have money”* (Shane, 65‐year‐old male).

Injecting behaviours were however viewed by some participants as crossing a line of acceptability, with one participant recalling that “*some people use heroin but they look down at people that inject heroin”* (Drake, 64‐year‐old male). This often led to feelings of shame or self‐blame which were often described in contrast to their ‘good’ upbringing.

*“Well, you know, like, I grew up, you know, in a good family. I grew up with morals and values, you know. And*, [with] *addiction, slowly but surely those morals and values get stripped away, you know. I've become morally bankrupt”* (Darko, 54‐year‐old male).

*“I allowed it into my life and I guess I'm now dealing with the consequences. But it's all me. I don't put it on anyone else. But I'm ashamed of myself. I'm saddened. I know I'm better than that. I had those choices. I knew the right answer but I still went there”* (Pam, 46‐year‐old woman).

Key themes emerging from the data were the middle‐class social position of the sample, their generally positive experience with, and uptake of, local services, access to drug supply through closed or restricted networks of acquaintances on The Beaches, and limited contact with the criminal justice system.

### 3.1 Social position

The majority of our interview sample reported coming from wealthy families (*n* = 16), many of whom owned property or were involved in family businesses. Others had trained and worked in professions, and one participant had competed as a professional athlete in a lucrative sport. While most of the participants did not describe having accrued significant wealth at the time of the interview, many came from upper middle‐class backgrounds, living in sizable family‐owned homes as they grew up, and with parents working in prestigious and well‐paying professions.

*“My father was a doctor, a surgeon actually and my mother was a nurse. He was also an academic*” (Drake, 64‐year‐old male).

*“My dad was an insurance broker…* [which] *was quite a lot of money”* (Pam, 46‐year‐old female).

Others grew up in more modest conditions, describing lower middle‐class childhoods. These participants were most often from migrant backgrounds and had parents that owned small, but successful businesses.

*“*[Mum] *owned her own shoe shop”* (Sandra, 50 year‐old female).

*“My parents, we've got a little fruit shop and a coffee shop*” (Joel, 48‐year‐old male).

While most participants described comfortable, middle‐class upbringings, this did not translate into personal wealth or affluence among any of the sample at the time of interview. The advantages of growing up in comfortable homes and neighbourhoods were described by participants as lingering material and social relations rather than economic wealth.

Two participants described difficult economic situations growing up, with parents living paycheck to paycheck or having to rely on government payments. However, both these participants reported that at least one parent had a history of drug dependence.

*“Primarily my dad having issues with finding rental properties and maintaining the payments and that sort of thing, so finances were a big issue. And also he's an ex user himself so he was struggling with his own recovery”* (Caroline, 28‐year‐old female).

*“I grew up pretty easy, it wasn't like I grew up without anybody or in a housing commission, but we didn't have money, like my dad and mum are both ex‐users”* (Seb, 30‐year‐old male).

The participants quoted above and one other were the only ones to report exposure to injection drug use in their childhood. At the time of interview only one participant was sleeping rough.

### 3.2 Service utilisation

Drug‐related stigma permeated accounts of health service engagement among our interview sample. Several participants recall perceptions and experiences of being mistreated by staff at mainstream health services, such as hospitals and by paramedics.

*“You notice even the fact they go put double gloves on … you know what I mean? You've probably definitely got a disease because you're a drug addict”* (Moran, 43‐year‐old female).

Despite this, our interviewees tended to maintain a generally positive attitude towards staff in these settings.

*“When I first came in, they just looked at me like you're a junkie and you're wasting our resources, it's your own fault. Once they got to know me, they're actually the nicest, and trying to get me to stay longer”* (John, 50‐year‐old male).

According to participants, instances of judgemental and discriminatory treatment were not the norm. When asked about the health and social services in the local area almost all participants were positive and indicated that such services were of ‘high quality’.

*“I have been to Northern Beaches Hospital. I've been to Manly Hospital. The services I think are much better over here. They're definitely—the quality is right up there”* (Moran, 43‐year‐old female).

In particular, they described local services as highly accessible.

*“People shouldn't be so scared to approach even for health advice. To get tests, that sort of thing, they shouldn't be so scared*.” (Caroline, 28 year‐old female)

*“It's all in great close proximity. And the one great thing about the Northern Beaches is that everything's at your—Centrelink is there, and then next door—upstairs is you've got your housing, and then you've got this new health facility”* (Jessie, 41‐year‐old female).

When talking about drug treatment services, the positive tone remained, with one participant saying of staff at the local methadone clinic: “*They're very lovely*” (Sandra, 50‐year‐old female). This affirming attitude was especially emphatic when participants were asked about local harm reduction services.

*“They're just incredible. And they're a really good service”* (Drake, 64‐year‐old male)

*“Awesome. Yeah, very good like, everyone's supporting”* (Joel, 48‐year‐old male).

*“I have a chat with a lady there* [local NSP], *usually, there's a particular lady on, and she's really nice, really helpful. She always asks me about how my life's going and stuff like that … Yeah, you don't feel judged or anything there”* (Busker, 49‐year‐old male).

*“I'm very happy with the needle exchange services and the drug and alcohol services here at* [Community Health Centre]*. I think that these public services, especially the needle exchange and free fits* [syringes] *is very good and healthy”* (Shane, 42‐year‐old male).

Despite experiencing some drug‐related stigmatisation by mainstream health services, participants did not indicate this was a deterrent to service utilisation. Overall, participants were very likely to report engaging with both mainstream and drug‐related health services when needed, and were particularly positive about local harm reduction services.

### 3.3 Acquiring drugs

While most of the sample indicated that they had acquired drugs from street‐based drug markets at some stage, this was not the case when they returned to, or resided in, the Northern Beaches. Interviewees indicated that when they live on The Beaches they acquired drugs through closed networks of friends and acquaintances.

*“I always buy off people who, and people that I'm generally friends with or whatever”* (Marianne, 24‐year‐old female).

*“You've got a phone number, word of mouth through other users. Networks”* (Joel, 48‐year‐old male).

These networks often stretched back to childhood and school, and the mainstream middle‐class party scene.

*“I hassled a mate for ages to do it for us … it was a mate from high school, actually”* (Slone, 50‐year‐old male).

*“Just word of mouth from a* [high school] *friend. And then I may get them to get it for me first, and they introduced me, so just like that”* (Moran, 43‐year‐old female).

They also extended to acquaintances made during the course of using and injecting psychoactive substances.

*“At first I was just the person hanging around with* [acquaintance name] *but that was years ago. Now I've got to know them all myself”* (Drake, 64‐year‐old male).

*“I just got introduced through a girl, she's introduced me and a lot of people from the Northern Beaches”* (John, 50‐year‐old male).

*“When you use heroin, it's word of mouth, I suppose … I've always had good contacts … Just like how everyone communicates, I suppose. Just call them.”* (Slone, 50‐year‐old male).

Transactions to acquire drugs on The Beaches therefore often involve direct contact with a person they know or who has sold to them in the past: *“Yeah, it was through people. You knew that he had it and you'd ring him and you'd go there”* (Darko, 54‐year‐old male). It was also common for the person selling the drugs to deliver directly to the home of the participant: *“They basically come to your house. They drop it off at your house”* (Moran, 43‐year‐old female). However, some reported transactions rarely occurred at the same place: *“All over the place, if they're wise, they'll keep moving around”* (Slone, 50‐year‐old female).

In addition to the absence of street‐based drug markets and unlike the drug scene in other areas of Sydney [31, 32], participants described drug transactions on The Beaches as relatively safe and cordial.

*“They're just normal people. They're just normal people that, maybe, are just doing it, selling for their own habit or whatever. But most of them are just normal people that are trying to get ahead themselves. And they were all really polite and decent to me”* (Sandra, 50‐year‐old female).

Also unlike the street‐based markets in the inner‐city and outer suburbs of Sydney [33, 34], the drug market on The Beaches involved a mixed group of both marginalised people who use drugs and middle‐class and decidedly wealthy people.

*“The quality* [of drugs] *has always been good up here … It's just the type of people that live up here … Rich people, you know, beach people”* (Shane, 65‐year‐old male).

*“There's a huge demand on the Northern Beaches for cocaine and opiates, there always has been, because as I said, people have the money here to afford cocaine … People pull up in BMWs, and ask me what's the heroin like at the moment”* (Sandra, 50‐year‐old male).

While these local networks are the first port of call for purchasing drugs when living on The Beaches, the closed nature of the network meant that it was not the most reliable market in which to acquire drugs—contacts did not always have what participants were looking for. Participants reported that when they could not access the drugs they wished to purchase through these networks, they resorted to street or other drug markets in the inner‐city and Western/South Western suburbs utilising knowledge and connections from both local networks and networks they had established when living in other parts of the city.

### 3.4 Criminal justice involvement

Only three participants in our study had spent time in gaol, mostly for offences they were ‘pinched’ for when living outside the Northern Beaches. However, many participants reported frequent encounters with police during periods of heavy use and frequent injecting in the inner‐city.

*“Alleyways in the back of the Cross, getting a flogging* [by police]*. Goes with the territory I suppose. What can you do, they've got a gun and a baton. Can't do anything”* (Slone, 50‐year‐old male).

*“I used to have some cops* [that] *really had it in for me but I was living pretty feral at the time. I was living on the* [inner‐city] *streets”* (Busker, 49‐year‐old male).

By contrast, when asked about their experiences with police when in the Northern Beaches, most participants indicated that they had very limited contact with police in the area.

*“I haven't got searched in the last two or three years now”* (Joel, 48‐year‐old male).

*“I don't really have run‐ins with the cops”* (Marianne, 24‐year‐old female).

*“Don't have any contact* [with police]*”* (Caroline, 28‐year‐old female).

*“They've always been really quite respectful with me”* (Pam, 46‐year‐old female).

Participants also indicated that they rarely, if ever, injected in public on The Beaches, even those who previously injected in public in other locations.

*“Just in my bedroom, or in the car, or at the dealer's house”* (John, 50‐year‐old male).

*“I'd have to be inside a house, yeah, definitely. If I was at a friend's place, I felt comfortable”* (Pam, 46‐year‐old female).

That does not of course mean that police contact did not occur on the Northern Beaches or that it was not targeted towards people who use or inject drugs. Indeed, a specific suburb was named by most participants as an area that police targeted people who use drugs. This suburb is known among participants (and other locals) as the ‘ghetto’ of The Beaches, described as the poorest part of an otherwise affluent area, which makes it ‘stand out’. Participants recalled a variety of overzealous forms of policing and surveillance of people who lived in or occupied this area.

*“I've lived in the area for over 28 years. So once you get pulled over, and they know you're on the methadone program, that tells them* [you] *have used heroin … So they might start following you in the car. So, my partner and I are often pulled over and have the car searched, and our bodies searched. I've been searched in the middle of the street, in front of, yeah, it's embarrassing”* (Sandra, 50‐year‐old female).

*“*[Officer in targeted suburb] *pull me up and he always used to get a towel around the door and made me strip in the middle of the street. Made me pull my pants down and if it didn't he'd fucking punch me in the face. He'd hold the towel and he'd just like punch you in the head, or he'd get the other cop and he'd hit you in the back with the taser so you fell to the ground anyway, and you're naked on the ground”* (Seb, 30‐year‐old male).

*“I remember being at the clothing bins one day in* [targeted suburb]*, and everyone rushed away. And I had a personal bit on me and a pipe. And they* [local police] *asked to go through my bag. And I didn't want them too. And I do believe you're allowed to deny them—well, they don't usually let you. And I'm thrown onto the ground”* (Jessie, 41‐year‐old female).

Even participants who reported minimal police contact recognised and sought to avoid the increased intensity of policing in this particular area, saying “*I stay away from* [targeted suburb]” (John, 50‐year‐old male) and warning friends that “*they're hot in* [targeted suburb] *tonight*” (Seb, 30‐year‐old male). However, police targeting of people who use drugs was, with some notable exceptions, largely restricted to this particular suburb rather than a characteristic of drug policing on the Northern Beaches generally.

## 4 DISCUSSION

Our study provides novel insights into the lives of people who inject drugs in an affluent community on the Northern Beaches of Sydney. In the first instance, the study reports on a sample that occupy a distinct social position, one which is rarely reported on in the literature. Our participants grew up in a range of middle‐class conditions, enjoying childhoods characterised by overt wealth and abundance, as well as more modest, but still comfortable circumstances. When compared to periods of living in the inner‐city and outer suburbs, or when visiting these areas to purchase drugs, interviewees described being less exposed to violence, whether structural or interpersonal. Beyond the distinct social position of our sample, we described patterns of drug use and acquisition, and documented differences in the way processes of marginalisation and criminalisation play out for our sample.

For instance, in the social science literature people who inject drugs often describe a tenuous relationship with service providers that was not experienced by those in our sample. Among people who inject drugs stigma and poor treatment from health service providers often dissuades health service engagement, along with difficulties, such as organising appointments and transport [35]. Though instances of stigmatising treatment were reported by our sample when they visited services, this was not the dominant experience, nor was it described as a deterrent to future service access. In the literature it is also common for people who inject drugs to experience a reduced sense of urgency in accessing drug‐related services due to more immediate concerns related to, for example, homelessness, taking priority [36, 37, 38, 39]. Only one person in our sample was homeless at the time of interview.

Another important point of comparison is that in much of the literature people who inject drugs utilise street‐based drug markets and are criminalised because of significant “fears around public and police detection” [40, p. 11] and “confrontational contact with the police” [41, p. 54]. People who inject drugs also report frequent police contact and experiences of re‐incarceration [42]. The opposite was true for our sample when living on The Beaches, and one of the potential reasons for this is the way our participants acquired drugs in suburban settings where no notable street‐based drug markets exist. Given that the research was conducted in a predominantly White and middle‐class area, our recruitment also reflected this resulting in a predominantly White and middle‐class sample, which likely impacted the comparative structural advantage outlined above. The differential and intersectional distribution of this structural advantage deserves a fuller analysis, but is beyond the scope of this paper.

Ethnographic research in the Lower East Side of New York City [43] which examined the relationship between suburban non‐injecting heroin users and the urban street‐based heroin market, found that those who lived in comparatively wealthier suburban areas tended to procure drugs through friends or intermediaries that distributed locally to a small number of people. This work also found that these low‐level sellers tended to be young and White, and catered only to other White, relatively affluent people for the purpose of profit and increasing beneficial social ties. This suggests that people from backgrounds and neighbourhoods of relative privilege may be more motivated and better able to limit and conceal their drug use, including not injecting drugs, and thus minimise its potentially damaging effects.

Our study extends this literature by providing a qualitative account of how these transactions and markets operate among people who inject in an affluent area of Sydney. While our participants had all accessed street‐based drug markets in the inner‐city, either during stages of their life where they lived there or as part of periodic visits to acquire drugs, when living in The Beaches they were most likely to access closed and personal networks for peer‐to‐peer drug transactions. This was made possible by a set of structural conditions, chief among them the minimal targeting of people who use drugs by police, which was limited to one suburb with a concentration of social housing. The absence of a street‐based drug market on The Beaches means that drug transactions can be assimilated into ‘non‐threatening' middle‐class suburban settings that are not regular targets for police surveillance. This includes anything from a café at the local shops to the parking lot of a large shopping centre, the front gate of a block of townhouses to the driveway of a modest suburban family home. Furthermore, this market caters to more than the desperate and drug dependent, with wealthy and occasional drug users forming a notable element of those who are part of closed networks for drug acquisition.

A final point of comparison between the literature and our study is the way participants viewed their drug use. Unlike many research participants recruited from the inner‐city and outer western suburbs, our interviewees drew on the language of recreational and functional drug use as a key frame of reference in describing their injection drug use. There is a small literature on functional non‐injecting drug use, which indicates that there are groups of people who use drugs occasionally and in controlled ways. This includes an Australian study of a group of drug enthusiasts who engaged in an annual carnivalesque recreational drug binge which acts as a parody of sober society and is dubbed “St. Oswald's Day” [44]. The functionalist drug use literature also explores the ways in which long term cannabis use is managed [45, 46, 47], ecstasy use is central to rave and club scenes [48, 49], normalised use of dexamphetamines in the pursuit of ‘controlled pleasure’ [50], how women harness the pharmacological properties of amphetamine‐type stimulants to meet the physiological demands of sex work in a context of limited economic opportunities [51] and the way that oral use of pharmaceutical opiates has become part of the lexicon of curious middle‐class drug use [41, 52].

While the participants in our study did not necessarily claim to be ‘functional’ users that held down consistent employment and remained closely linked to mainstream society, they did describe their use in ways that emphasise controlled consumption [53, 54, 55]. Examples include the way interviewees report avoiding use and intoxication in front of family members, avoiding daily use or only injecting occasionally as a ‘treat’. This mirrors findings about the way that people who use opioids orally in affluent areas of Sydney engaged in research‐informed self‐limiting behaviours to avoid stigma [41]. By contrast, participants in our sample described managing their drug use to maintain social status, sustain family relationships – who also provide financial support—and to maintain a stable housing situation while they live on The Beaches. Importantly, such practices are made possible by the structural conditions of this suburban, beach‐side enclave. While all participants lived on the Northern Beaches at the time of interview, many had lived in other parts of the city when they first began injecting drugs or during periods of intensified drug use. Participants often move back to The Beaches to limit their injection drug use by living in the family home, staying in easier to access and ‘better’ quality social housing and avoiding police attention.

Finally, our findings also contribute to research about the role and purpose of pleasure in drug use. Our participants negotiated the perception that intoxication is harmful and the pleasure they derive from it [56, 57] by seeking to keep their drug use private and frequently invoking notions of self‐control [58] in explanations about the pleasure of injection drug use. Participants also spoke of drug use as ‘controlled release’ [59] that formed part of part of lifestyles of freedom, exploration and pleasure among social circles in the mainstream drug scene [60, 61], in the Northern Beaches [41], and about cultivating ‘subcultures’ of this that involved injection drug use. Our participants described the reasons for injection drug use as similar in many ways to other forms of recreational drug use that occurred on The Beaches, as well as evolving out of the similar networks of people who use, share and sell drugs.

## 5 CONCLUSION

Consistent with the literature our study provides further evidence of the ways in which social, economic and structural conditions, and variations in the degree or level of stigma and discrimination, impact the marginalisation and criminalisation experienced by those who inject drugs. In our study however, rather than occurring in the underbelly of the inner‐city or being defined by narratives of marginalisation and disadvantage, our findings have provided a qualitative articulation of injection drug use that is occurring in the ostensibly comfortable, middle‐class environments of everyday life in the suburbs. The study provides rare insights into the lives of people who inject drugs in an affluent community. The Northern Beaches is synonymous with a lifestyle of freedom and abundance, one where drug use complements an orientation towards relaxed, social lifestyles and accommodates curious and playful experimentation with drugs [41]. In this setting the drug market operates covertly as part of the normal rhythms of middle‐class life, in among the bustle of cafés and shopping centres, or in the context of a friendly neighbourhood chat on the driveway or doorstep. Injection drug use is largely hidden too, occurring in the privacy of people's homes, where police rarely venture. Our study also extends the field's understanding of intermittent, self‐limiting and functional drug use and provides evidence of the way structural conditions in affluent communities can limit the marginalisation and criminalisation experienced by those who inject drugs.

## AUTHOR CONTRIBUTIONS

The first author coneptualised the project, conducted the feildwork, analyised the data and drafted the manuscript. The second author analysed the data and reviewed manuscript drafts. The third author provided overall guidance of the project, including assisting in the conceptualisation of the project, critical reflection during feildwork and the review of manuscript drafts.

## CONFLICT OF INTEREST

The authors declare that there is no conflict of interest.

## ETHICS STATEMENT

Ethics approval for the study was received from the UNSW Sydney Human Research Ethics Committee and participants were remunerated $AUD50. All interview participants nominated or were allocated a pseudonym to be used in any dissemination of study results.
